# The Beta Amyloid Dysfunction (BAD) Hypothesis for Alzheimer’s Disease

**DOI:** 10.3389/fnins.2019.01154

**Published:** 2019-11-07

**Authors:** Heinz Hillen

**Affiliations:** Independent Researcher, Haßloch, Germany

**Keywords:** amyloid beta protein, therapy, Alzheimer’s disease (AD), vaccination, hypothesis

## Abstract

Beta amyloid, Aβ 1–42, originally named as Amyloid A4 protein, is one of the most investigated peptides in neuroscience and has attracted substantial interest since its discovery as the main insoluble fibril-type protein in cerebrovascular amyloid angiopathy (Glenner and Wong, 1984; Masters et al., 1985) of Alzheimer’s disease (AD). From the very beginning, Aβ was regarded *per se* as a “bad molecule,” triggering the so-called “beta amyloid cascade hypothesis” (Hardy and Higgins, 1992). This hypothesis ignored any physiological function for *in situ* generated Aβ monomer with normal production and turnover rate (Bateman et al., 2006). Accordingly, pan-Aβ-related therapeutic approaches were designed to eliminate or lower the three structural isoforms in parallel: (1) the pre-amyloid monomer, (2) the misfolded oligomer, and (3) the final fibril. While we already knew about poor correlations between plaques and cognitive decline quite early (Terry et al., 1991), data for an essential benign physiological role for Aβ monomer at low concentrations were also not considered to be relevant. Here, a different Beta Amyloid hypothesis is described, the so-called “Beta Amyloid Dysfunction hypothesis,” which, in contrast to the “Beta Amyloid Cascade hypothesis,” builds on the homeostasis of essential Aβ monomer in the synaptic vesicle cycle (SVC). Disease-relevant early pathology emerges through disturbance of the Aβ homeostasis by so far unknown factors leading to the formation of misfolded Aβ oligomers. These early species interfere with the synaptic physiological Aβ monomer regulation and exert their neurotoxicity via various receptors for sticky oligomer-type Aβ aggregates. The Beta Amyloid Dysfunction (BAD) hypothesis is introduced and shown to explain negative clinical results of Gamma-secretase and Beta-secretase (BACE) inhibitors as well as pan-Aβ isotype unselective immunotherapies. This hypothesis gives guidance to what needs to be done therapeutically to revive successful clinical testing in AD for this highly validated target. The BAD hypothesis will need further refinement in particular through more detailed exploration for the role of physiological Aβ monomer.

## Introduction

Aβ and in particular Aβ1–42 so far has been the main target molecule pursued by the pharmaceutical industry to achieve disease-modifying treatments in Alzheimer’s disease (AD). Genetic exploration of its amyloid precursor protein (APP) (Dyrks et al., 1988) and processing by Beta-secretase (BACE; Vassar et al., 1999) and Gamma-secretase complexes PSEN1 and PSEN2 (Wolfe et al., 1999) have led to a basic molecular understanding of its bioprocessing.

Mutations in PSEN and APP genes in rare cases of Familiar Alzheimer’s Disease (FAD) (St George-Hyslop and Petit, 2005) either result in aberrant amyloid beta production or lead to an unfavorable shift in Aβ40/Aβ42 ratio and in some cases to increased Aβ production (Weggen and Beher, 2012). Altogether, the variety of different mutations give substantial evidence for the validation of Aβ as a valuable target in AD.

A long series of clinical disappointments starting with an aggregated Aβ AN-1792 Active Immunization study (Bayer et al., 2005; Nicoll et al., 2019) in 2001 up to recent failures of various BACE inhibitors (Panza et al., 2018; Egan et al., 2019) and two gamma secretase inhibitors (Coric et al., 2012; Doody et al., 2013) have created substantial doubts about the underlying Amyloid Cascade hypothesis (Hardy and Higgins, 1992). From the protagonists of the Cascade hypothesis, the most obvious reason for clinical failure could be the very early onset of Aβ pathology. This may only be partly true, since it does not explain the shared cognitive side effects for structurally diverse Gamma- and beta-secretase inhibitors in clinical testing. The main discussion here is focused on limitations of individual clinical candidates rather than questioning the validity of the underlying hypothesis. The majority of scientists still look at “Amyloid beta” as a homogeneous target and have not incorporated the variety of biology between the *in vivo* existing three major and very different protein assemblies, all of them named “Aβ,” albeit they only share the primary amino acid sequence. These are (1) Aβ monomer, (2) misfolded soluble Aβ oligomers, and (3) fibrillar Aβ.

The Aβ field has suffered from a persisting undistinguished isotype terminology in most scientific papers. In order to enable a precise understanding of the new Beta Amyloid Dysfunction (BAD) hypothesis, this article will try to clarify upfront some definitions on the three different “Aβ” forms.

## Definitions of Aβ Isoforms

**FIGURE 1:**
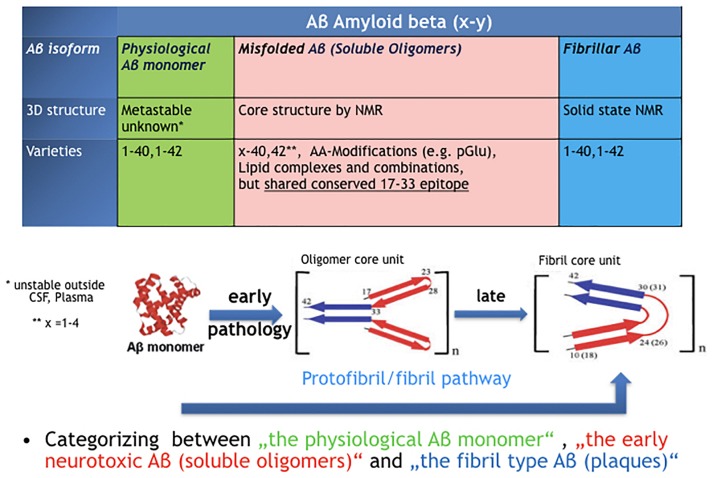
The confusion around the peptide “Amyloid beta, Aβ.” Three very different types of protein assemblies sharing the same name.

Figure 1 illustrates the three fundamental structural forms that are known for “Aβ” and key features we know in terms of structural core elements characteristic for oligomer- and fibril-type Aβ.

### Aβ Monomer

Aβ is generated in synaptic processes at relatively high production and turnover rates (Bateman et al., 2006). It is released as Aβ monomer by activated neurons (Cirrito et al., 2008) and has been shown to modulate synaptic activity in a time- and concentration-dependent manner (Kamenetz et al., 2003; Abramov et al., 2009). Aβ dynamics correlate with neurological status in acute brain injury (Brody et al., 2008). From a physicochemical perspective, the Aβ monomer belongs to the class of intrinsically disordered proteins (IDP) that lack a fixed three-dimensional structure (Oldfield and Dunker, 2014). Aβ is metastable in aqueous solutions and gains stability by forming secondary structure in membrane-like environment (Morgan et al., 2004). Hence, unlike its precursor APP, we do not have solid experimental data on instable nascent Aβ monomer in particular outside the CSF compartment. It is suggested that Aβ monomer Aβ1–40 and Aβ1–42 upon well-controlled release by BACE- and Gamma-secretase are essential facilitating molecules to contribute to synaptic vesicle cycling in neurons (Cirrito et al., 2008; Ovsepian et al., 2018). CSF Aβ is almost perfectly monomeric, since oligomeric and fibril-type Aβ species are below detection limits of conventional bioassays (Rosén et al., 2013). Recently, ultrasensitive tests have been described that claim detection of aggregated Aβ in human CSF of AD patients, but these are not fully validated (Salvadores et al., 2014; Yang et al., 2015) so far. Although monomeric Aβ and in particular Aβ1–42 is highly prone to aggregate, folding control through chaperones normally guarantees a precise control of biosynthesis and physiological turnover in non-diseased young and most older human beings. Significant levels of aggregated protein species with high molecular weight would create a substantial increase in viscosity and could not be tolerated in thin CSF. Decreasing CSF Aβ 42 monomer levels is one of the earliest and best biomarker for patients with mild cognitive impairment (MCI) or AD.

### Misfolded Aβ Oligomers

Misfolded Aβ oligomers are defined as any Aβ species that undergoes structural transformation into at least dimers or multimers, thereby changing its 3D structure into detergent-soluble amyloid Aβ species. While nascent monomers in healthy individuals undergo complete physiological processing, oligomers bind to a number of proteins and receptors (Cline et al., 2018). The common denominator for all early Aβ oligomers Aβx-y is a characteristic epitope in the middle part of the peptide (AA17–AA33). This epitope is different in fibril-type Aβ and not available in nascent Aβ monomers (Yu et al., 2009). The definition by a common epitope is more helpful compared to a classification by size or peptide modification (Aβx-y)_n_. Following the epitope definition, smaller fragments with a fibril-type core structure should not be included here and named as preforms of fibrils, i.e., protofibrils. Though in many papers the name protofibrils is often used as synonym for Aβ oligomers which leads to confusion. The majority of AD brain deposited Aβ oligomers can be precisely characterized next to monomer and fibrils through immunoprecipitation by immobilized Aβ oligomer isotope selective antibodies from AD brains and subsequently in a separate step quantified independently as Aβx-y oligomers (Hillen et al., 2010).

In contrast to fibril-type Aβ, all misfolded Aβ oligomers from *in vivo* brains dissolve in SDS-containing buffers.

It should also be mentioned here that every monomeric protein displays some minimal reversible equilibrium with its non-misfolded dimeric form. Due to this effect, in some test settings, e.g., like symmetric ELISAs, Aβ oligomer data might be misinterpreted in terms of detection of traces of misfolded Aβ in human fluids, like CSF or plasma.

### Aβ Fibrils

Aβ fibrils were the first Aβ isoform discovered as “Amyloid beta, A4” in brains of AD patients (Glenner and Wong,1984). The regular structure of Aβ fibrils is well defined and stable (Petkova et al., 2002). Fibrils can nicely grow by adding Aβ monomer but not by adding Aβ oligomers (Gellermann et al., 2008), indicating that the oligomers are not direct intermediates on the folding pathway of fibrils. Fibril-type structures are clearly distinguishable from oligomer-type Aβ via solid-state NMR or by highly oligomer selective antibodies (Yu et al., 2009; Hillen et al., 2010; Lu et al., 2013).

Fibrils are the main Aβ form in plaques and, in contrast to detergent-soluble Aβ oligomers, can only be dissolved in formic acid.

## The Beta Amyloid Dysfunction Hypothesis

### Aβ Monomer Homeostasis Is Essential for Synaptic Function

Aβ biogenesis and turnover is an essential physiological process conserved in neurons of vertebrates. The central point of difference between the old Aβ Cascade hypothesis and the new Dysfunction hypothesis is the claim for a physiological role of the Aβ monomer in non-diseased individuals. There is multiple evidence that Aβ monomer plays an important role in synaptic activity regulation (Cirrito et al., 2005; Abramov et al., 2009; Morley et al., 2010). The group of Copani has suggested that the “loss-of-function” hypothesis for the role of Aβ in neurons should be taken into consideration (Giuffrida et al., 2009). The homeostasis of synaptic Aβ monomer levels is critical and extremely complex. The complexity of Aβ monomer physiology is due to the interplay of a metastable hydrophobic peptide with membranes, lipids, and vesicles. Quantifying metastable Aβ monomer concentrations in living organisms is challenging since IDPs can adopt their structure upon extraction. While there has been some success with a combination of synchrotron-based Fourier transform infrared micro-spectroscopy and non-denaturing gel electrophoresis to characterize intermediate Aβ species in APP TG mice (Klementieva et al., 2017), it remains extremely difficult to determine nascent Aβ monomer concentrations *in vivo*.

It can only be postulated that Aβ pathophysiology is assumed to start, when the formation and metabolism process is disturbed. Table 1 summarizes key papers supporting the physiological Aβ role.

**TABLE 1:** Key findings to the essential role of Aβ monomer in physiological synaptic processes.

**Item**	**Key data**	**Paper**
Synaptic processes	• Endogenously released Aβ peptides regulate synaptic transfer at single pre-synaptic terminals and synaptic connections in rodent hippocampal cultures and slices	Abramov et al., 2009
Synaptic activity	• Inhibiting neuronal activity by TTX decreases ISF Aβ levels	Cirrito et al., 2008
Learning and memory	• Low doses of icv administered monomeric Aβ improves cognitive behavior in non TG mice	Morley et al., 2010
	• Anti monomer Aβ antibody impairs cognitive behavior in non TG mice	
	• Aβ monomer is essential for LTP	
	• Impaired learning by anti sense Aβ	
Cortical neurons	• Aβ production is critical for viability of neurons	Plant et al., 2003
	• Aβ monomer is neuroprotective	Giuffrida et al., 2009
APP KO mice	• Impaired LTP	Dawson et al., 1999
	• Poor performance in spatial memory tasks	andSeabrook et al., 1999
	• Reduced synapses	
PS1 deficient mice	• Reduced Aβ levels	Morton et al., 2002
	• Impaired LTP	

Impairment of the Aβ turnover process results in early generation of misfolded neuropathogenic species on cost of synaptic physiological Aβ levels. The BAD hypothesis of AD predicts that both effects synergize and contribute to early pathology via two mechanisms: Aβ oligomer-type neuropathogenic species bind to various receptors mediating cell toxic effects (Lauren et al., 2009; Ohnishi et al., 2015) and secondly disturbance of Aβ monomer homeostasis at synaptic sites through Aβ oligomer formation. If we consider optimal monomer concentration at the synapse to be a critical factor, replenishment upon loss of Aβ monomer is immediately needed. This can only be achieved by increased production of Aβ via APP processing and BACE up-regulation. Through this positive feedback loop, an additional risk of misfolding by enhanced Aβ monomer production and turnover is created. The best indicator for this effect is elevation of BACE in CSF of AD patients (Zetterberg et al., 2008; Decourt and Sabbagh, 2011) as well as in pre-synaptic terminals of TG models of AD (Zhang et al., 2009).

Early biochemical deviations from Aβ physiology in MCI are indicated by lowered CSF Aβ42 monomer already before substantial loss of neurons occurs in later stages of AD (Fagan et al., 2009).

Monomeric Aβ can be regarded as a benign pre-amyloid peptide that serves as a permanent source for an essential physiological cofactor needed in the synaptic vesicle cycle (SVC) process (Südhof, 1995). The SVC is known to be the primary site for Aβ production enriched in pre-synaptic terminals (Thinakaran and Koo, 2008; Müller et al., 2017). So far, the data for potential Aβ monomer involvement in SVC’s turnover were discussed either as contributions by the precursor molecule APP or in the context of pathophysiology only. In analogy to research on synuclein, where the molecular differences between physiological and pathophysiological isoforms are better understood (Burré et al., 2014), we still need more efforts to explore the details of a potential physiological role for Aβ monomer in binding proteins in the course of synaptic vesicle formation and exchange.

Once we have agreed to claim a physiological role for Aβ monomer and to categorize the Aβ structural isoforms in the three different buckets Aβ monomer, early misfolded Aβ oligomers and Aβ fibrils, the next step is to look at how these forms in terms of physiology and pathophysiology are connected and interdependent. In the next four paragraphs, the fundamental concept of the BAD hypothesis will be laid out.

### Aβ Bioprocessing in Phases of Physiology, Pathophysiology, and Therapeutic Recovery

#### Part 1. The Physiological Aβ Production and Turnover

**FIGURE 2:**
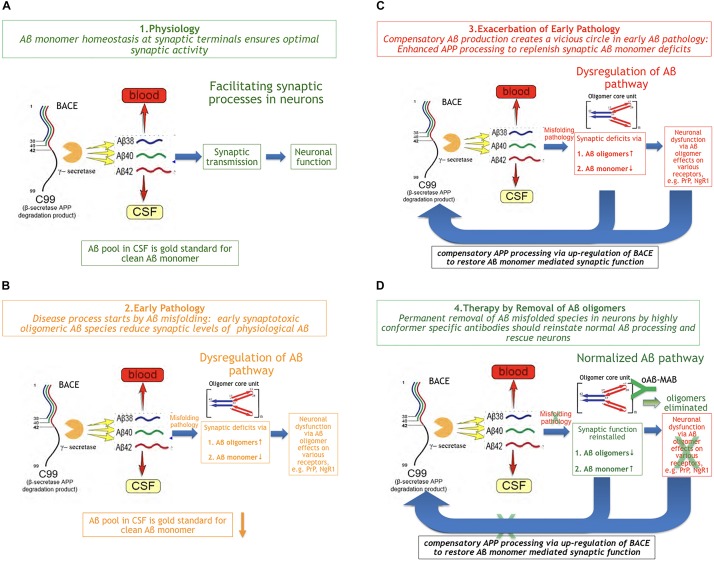
The Beta Amyloid Dysfunction Hypothesis (BAD). **(A)** Part 1. The Beta Amyloid physiological process [fragments of graphics in panels **(A–D)** were taken from Patterson et al. (2015)]. In normal individuals, Aβ monomer is produced, metabolized, and replaced at high rates without formation or residual aggregates. **(B)** Part 2. The Beta Amyloid pathophysiological process begins. Aβ monomer folding control is becoming imperfect and Aβ oligomers begin to form on cost of monomer concentration at synaptic sites. While, in FAD, exhaustion of chaperones can be assumed as the main trigger for pathology, the reason in sporadic AD is unknown so far. **(C)** Part 3. Exacerbation of pathology through positive feedback loop. In order to maintain Aβ monomer homeostasis, the loss of Aβ monomer induces increased Aβ production by BACE up-regulation, which exacerbates Aβ misfolding rate: a vicious cycle. **(D)** Part 4. Aβ oligomer selective immunotherapy reverses Aβ dysregulation by removal of misfolded species (oAβ-MAB = selective anti Aβ oligomer monoclonal antibody).

There is substantial evidence about a physiological role of Aβ monomer summarized and reviewed by Copani (2017). Aβ production and turnover have been determined in human CSF *in vivo* by 7.6 and 8.3%/h, respectively (Bateman et al., 2006). These values are exceptionally high and reflect the challenge for a difficult protein to be maintained in a correct shape under folding control *in vivo*. Using the Stable Isotope Labeled Kinetics (SILK) technology, Bateman has demonstrated that Aβ turnover is changed in AD and slows down by age about 2.5 fold (Patterson et al., 2015). Aβ concentrations are highly regulated at synaptic sites (Figure 2A). This comprises the well-controlled Gamma- and beta-secretase activities that together ensure optimal synaptic Aβ monomer concentrations according to the individual neuron activity needed.

#### Part 2. The Early Pathophysiological Aβ Process

It is common knowledge now that Aβ pathology based on misfolding in brains precedes cognitive deficits in AD by more than a decade (Blennow et al., 2015). The earliest measurable Aβ parameter indicating Aβ pathology in MCI and AD patients is a lowered Aβ monomer1–42 CSF level. This decrease of the earliest Aβ marker reflects the start of an impairment in physiology, at a time when Aβ monomer escapes at least partly the *in vivo* folding control. While, in FAD, it can be assumed that chaperone control will be exhausted through APP mutations or overproduction at a certain point in time, it is unknown what triggers the beginning of oligomer formation in sporadic AD. In the early phase of MCI, Aβx*-*y oligomers with a characteristic structure on the typical oligomer folding pathway (Yu et al., 2009; Hillen et al., 2010) can be detected by oligomer selective antibodies (Figure 2B). These sticky molecules can bind promiscuously to many proteins and cell surface receptors, e.g., the PrPc protein (Smith and Strittmatter, 2017) or RAGE (Yan et al., 1996). Oligomers also impair the high neuronal energy consumption by binding to key mitochondrial proteins (Reddy and Beal, 2008). The deleterious effects of Aβ oligomers have been widely described and reviewed in the last two decades (Cline et al., 2018).

#### Part 3. Compensation Worsens Pathology

Besides the synaptotoxicity of oligomers via potential receptor-mediated effects, early Aβ monomer misfolding leads to a deficiency of Aβ monomer at the synapse. Assuming that neuronal activity-associated Aβ monomer concentration is an essential factor in modulating synaptic activity means to demand a compensation for Abeta monomer losses by BACE up-regulation. This could happen in a typical positive feedback loop. As a consequence of increased Aβ production, the misfolding rate continues to increase (Figure 2C). Hence, a vicious cycle of enhanced Aβ production and turnover exacerbates the Aβ pathology via increased oligomer formation and monomer deficiency in parallel.

Beta-secretase up-regulation upon Aβ monomer deficiency is not confirmed directly by data so far. But there is quite some evidence that BACE regulation is an extremely sensitive and early process tightly linked to pre-synaptic APP regulation. Like Aβ monomer reduction in CSF, increase of BACE1 activity is a very early biomarker of Aβ pathology in MCI and early AD (Zhong et al., 2007). BACE1 gene expression is elevated in sporadic AD (Fukumoto et al., 2002; Holsinger et al., 2002; Li et al., 2004). Synaptic BACE1 is colocalized with APP in the same vesicle (Steuble et al., 2012; Deng et al., 2013).

Substantial evidence for an existing feedback loop between Aβ oligomers and Aβ regulation is derived from oligomer binding to Nogo-66 receptor1 (NgR1), which has been shown to regulate APP processing (Smith and Strittmatter, 2017).

#### Part 4. Early Aβ Oligomer-Type Therapy Reverses Aβ Dysregulation

Following this concept, the therapeutic strategy should be reversing the pathology by removing the misfolded Aβ species in time *in situ* at neuronal sites as illustrated in Figure 2D. The most specific way of doing this would be a potent and sufficiently CNS-penetrant Aβ oligomer-directed selective immunotherapy by an individual antibody or a corresponding Active Immunization regimen. This should normalize Aβ turnover and consequently the biomarkers Aβ42 and BACE in CSF are expected to normalize as well. A potential co-therapy would be a combination with a pure gamma secretase modulator, to reduce Aβ aggregation propensity by increasing Aβ38 and Aβ40 on cost of Aβ42 levels.

#### The Pathology Starts in the Neuron

It is often argued that Aβ is an extracellular protein and cannot contribute to Aβ oligomer pathology inside the neuron. But there is multiple evidence that soluble misfolded Aβ species accumulate in the cell and disturb the physiological Aβ processing (Philipson et al., 2009; Youmans et al., 2012; Antonios et al., 2013; Zhao et al., 2014). The evidence for intraneuronal Abeta pathology is an additional argument for the interference of Aβ oligomers with potential Aβ monomer-mediated SVC formation and exchange processes in neurons.

## Discussion

### Cascade Hypothesis Versus Dysfunction Hypothesis

The BAD hypothesis is able to explain findings that are left unexplained by the Cascade hypothesis. Table 2 summarizes key major items that follow the rules of the BAD hypothesis but not the Cascade hypothesis.

**TABLE 2:** BAD vs. cascade hypothesis: key differences.

**Item**	**Beta amyloid cascade hypothesis**	**Beta amyloid dysfunction (BAD) hypothesis**
Physiological Aβ monomer	No function; source for aggregated Aβ	To be preserved at adequate concentrations in synaptic processes
Pathophysiology	Amyloid deposits (protofibrils, fibrils)	Misfolded species formed in neurons interfere immediately with default Aβ processing
Reduced Aβ CSF levels in AD	Difficult to explain	Benign CSF Aβ monomer inversely correlates with early neurotoxic Aβ oligomers
APP KO mice	Age dependent learning deficits indicate that ***sAPP*** is essential in synaptic processes	Age dependent learning deficits indicate that ***Aβ monomer*** is essential in synaptic processes
Genetic AD link in FAD	Not all PSEN mutations explained by Aβ overproduction	All PSEN and APP mutations interfere with homeostasis of regular physiological *A*β monomer

The plausibility for a physiological role of a highly expressed and high-turnover pre-amyloidogenic protein like the Aβ peptide is already given through analogy with other CNS proteins like synuclein and tau, which are known to have a structural isoform associated with a benign physiological function (Guo et al., 2017; Burré et al., 2018). According to the Cascade hypothesis, the Aβ formation is regarded as a superfluous relic left by evolution. But Aβ homeostasis in brains is an extremely well-regulated and energy-consuming process in normal human brains. Eliminating the generation of this central protein in AD therapy therefore seems to lack plausibility.

Another strength of the BAD over the Cascade hypothesis is that it defines the critical neurotoxic Aβ species as a molecule deviating structurally from the monomer in its earliest point in time, which does not leave the toxicity to very lately formed and highly insoluble inert fibril species in plaques. This concept is in line with observations that, (1) Aβ pathology and oligomer formation is detected very early and (2) plaques do not well correlate with AD. According to the BAD hypothesis, reduced monomeric CSF Aβ is the earliest indicator of started misfolding of Aβ in neurons. Finally, misfolded Aβ oligomer toxicity is not dependent on massive tissue accumulation anymore but becomes effective as early as physiological Aβ metabolism is disturbed at synaptic processes. The BAD hypothesis is compatible with all types of PSEN and APP-derived FAD mutations, which lead to either misregulation of Aβ production and turnover, or an aberrant Aβ molecule, like Arctic mutation E22G (Pifer et al., 2011).

### Therapeutic Implications

The advantage of the new BAD hypothesis in AD is to explain all major negative clinical results so far, while not restricted to the argument of too late treatment only. But more importantly, it guides into encouraging new future strategies.

Let us look into the unsuccessful history first. Table 3 summarizes predictions for clinical results following the two individual hypothesis.

**TABLE 3:** BAD vs. cascade hypothesis: prediction of clinical results for major therapeutic classes.

**Therapeutic class**	**Beta amyloid cascade hypothesis**	**Beta amyloid dysfunction (BAD) hypothesis**
Gamma-secretase inhibitors;	*Efficacious*	*Not efficacious*
BACE inhibitors;	Lower aggregation propensity	Inferior with role of physiological Aβ in synaptic function; risk of cognitive deficits through lack of physiological Aβ monomer at synaptic sites
Pan Aβ immunotherapy		
Gamma-secretase modulator	*Partially efficacious*	*Efficacious*
	Insufficient target coverage	Reducing aggregation propensity while maintaining overall Aβ monomer concentration
Early misfolded Aβ oligomer specific immunotherapy	*Partially efficacious*	*Efficacious*
	Insufficient target coverage	Neutralizing low fraction of pathogenic misfolded Aβ species generated during early impaired neuronal Aβ processing is sufficient

### Why Secretase Inhibitors Failed in Clinical Trials

The concept behind secretase inhibitors is to lower Aβ production, thereby indirectly avoiding amyloid plaque formation. Two Gamma secretase inhibitors, semagacestat (Doody et al., 2013) and avagacestat (Coric et al., 2012), and at least three beta-secretase inhibitors, verubecestat (Egan et al., 2019), atabecestat (Timmers et al., 2018), and lanabecestat (Cebers et al., 2017), have been clinically tested. Two of them have not only found to be inactive but to create cognitive impairments. So far, the reasons for cognitive side effects have been discussed as individual structural issues caused by the compounds or by target-related side effects. In the case of semagacestat, the inhibition of Notch and the accumulation of the beta C-terminal fragment were claimed to be responsible for cognitive side effects (Doody et al., 2013). For BACE 1 inhibitors, it has been suggested that the synaptic plasticity via seizure protein 6 (SEZ6) is a possible reason for cognitive impairment (Zhu et al., 2018). According to the BAD hypothesis, it is an expected result as a consequence of chronic Aβ monomer deficiency at synaptic sites. Following the BAD hypothesis, a Gamma secretase *modulator* rather than an inhibitor would be a possible treatment option, based on the fact that a decreased Aβ42/Aβ40 ratio would lower the aggregation propensity, while essential overall Aβ concentration could be maintained.

BACE elevation was always discussed as initial part of the pathology. It was not considered to be a consequence of a compensatory feedback. Following the BAD hypothesis, BACE is a good biomarker for Aβ pathology, but not a target for AD treatment.

### Why Aβ Monomer-Directed Immunotherapies Failed in Clinical Trials

Except for aducanumab (Sevigny et al., 2016), so far all clinically tested anti Aβ antibodies engaged the excess Aβ monomer. This violates the BAD hypothesis and should not contribute to tackle pathophysiology in AD. On top of this hypothesis, also from a pharmacokinetic perspective, pan-Aβ immunotherapy is practically impossible, since in the presence of excess Aβ monomer in plasma and CSF *in vivo*, these antibodies would need unrealistic high doses to neutralize minor oligomer fraction next to high concentrations of monomer at neuronal synaptic sites. Even in clinically established antibody treatments for peripheral diseases, practical doses of therapeutic antibodies can only deal with low concentrations of proteins, e.g., interleukins, which persist in fairly low tissue and plasma concentrations even under pathophysiological conditions. An attempt to profile the monomer and oligomer binding antibody crenezumab (Adolfsson et al., 2012) toward oligomer binding by raising the dose fourfold in Phase 3 after unsuccessful Phase 2 study was obviously not sufficient and failed. Hence, the oligomer hypothesis so far has not been tested by crenezumab.

### Why Aβ Fibril-Directed Immunotherapies Failed in Clinical Trials

Fibril-type Aβ in AD brain plaques are very late insoluble deposits formed by either addition of Aβ monomer to a template or potential slow conversion of Aβ oligomers. In any way, plaque load is not correlating well with cognitive impairment in AD (Terry et al., 1991; Aizenstein et al., 2008; Malek-Ahmadi et al., 2016). Therapeutically, solubilizing plaques also means to resolubilize inert fibril-type Aβ with possible transient creation and release of soluble oligomers at unknown concentrations and sites and potential implications for the AD pathology.

A recent paper in 2019 has followed surviving AD patients who underwent dissolution of plaques in the course of the AN1792 active immunization study (Nicoll et al., 2019). They did not show significant cognitive improvement. While part of the reason might be late treatment, it can be assumed that the polyclonal antibody response elicited by aggregated Aβ was Aβ isoform unspecific.

### Aβ Oligomer Neutralizing Treatments and Biomarker Options

Future clinical candidates for prevention and treatment of AD should not only display high potency for all early misfolded Aβx-y oligomers, but also demonstrate at least 1000-fold selectivity over Aβ monomer and Aβ fibrils. The best way to characterize potency and selectivity of candidate antibodies is immunoprecipitation of the majority of all Aβx-y species of misfolded and modified oligomers from post mortem AD brains under detergent and non-detergent conditions. Such antibodies have been described, e.g., 4D10 and similar globulomer-derived antibodies (Hillen et al., 2010; Barghorn et al., 2011, 2012). Other groups describe the generation of Aβ oligomer selective antibodies obtained by immunization with loop peptides (Gibbs et al., 2019).

Past clinical Aβ projects were often selected and designed along the availability of target engagement biomarkers, which enabled go/no-go decision points after a short proof-of-concept study. Clinical studies with secretase inhibitors have generated conclusive results, since *in vivo* potency was clearly demonstrated by dose-dependent significant reduction of Aβ levels in CSF. Future Aβ-related therapies in AD need to be tailored to clear exclusively pathophysiology while seeking new opportunities for early target engagement biomarkers. The globulomer technology has also provided an active vaccination regimen using a third-generation truncated globulomer mutant as antigen (Barghorn et al., 2016). The elicited polyclonal antibody response by these antigens is remarkably oligomer epitope specific.

An early biomarker concept to enable robust dose finding for Aβ oligomer antibodies might be more challenging since Aβ oligomers in CSF are, if at all, only present at extremely low concentrations. But *in vivo* Aβ kinetics measurements generated by clinical SILK technology (Patterson et al., 2015) offers an opportunity since it was shown to be dependent on age and AD status of patients and control subjects. In a clinical proof-of-concept study, potent Aβ oligomer selective antibodies should normalize Aβ metabolism also in an acute or subacute setting using SILK. This approach could serve as a dose finding study to de-risk a subsequent pivotal 18-month study.

The BAD hypothesis also predicts BACE levels to be normalized in CSF as well as Aβ monomer upon treatment. Proof-of-concept studies can even be back-translated into APP TG mice, since BACE activity and substrates were shown to be technically measurable in CSF of mice (Dislich et al., 2015).

Following the BAD hypothesis, it will also be possible to select and optimize the best anti-Aβ oligomer antibody treatments in AD. A typical iterative procedure would be screening of antibodies in emerging ultrasensitive Aβ oligomer CSF and blood tests. Those Aβ oligomer selective antibodies that score best in neutralizing traces of residual Aβ oligomers in brain extracts and CSF (Salvadores et al., 2014) should be preferred clinical candidates.

### The BAD Hypothesis Needs Further Refinement

The newly introduced BAD hypothesis in AD is meant to start a fundamental re-thinking for future Aβ-related treatments. After clinical failure of major amyloid beta treatments, for the majority of scientists, investors, society, and patients, the Aβ approach seems to be without a further perspective. Many companies and research institutions are turning away from the best validated target in AD without analyzing their basic mistakes in past strategies. Unfortunately, almost all clinical Aβ-related approaches so far seemed to be wrong, since they were targeting predominantly the physiology rather than the early pathophysiology of this challenging peptide.

The BAD hypothesis should encourage us to restart the clinical research with very different Aβ approaches and immunotherapies in order to profile highly oligomer selective and potent treatments, which have already been preclinically described (Hillen et al., 2010; Barghorn et al., 2011, 2012, 2016).

The BAD hypothesis will gain further validity when the exact role of a steadily produced Aβ monomer will be fully explored at the molecular level. Like for Tau and Synuclein, there should be a clear function for the highly regulated nascent Aβ monomer protein in the physiology of neuronal processes (Copani, 2017).

## Conclusion

Until we know more about the molecular mechanisms for the involvement of Aβ monomer in the neuronal SVC process, the current BAD hypothesis will help to guide the discovery, selection, and development of various Aβ oligomer-targeted drugs.

The holy grail will be a protective active vaccination regimen based on a surrogate antigen at minimal frequency, which elicits permanent specific humoral immune response directed to the early key misfolding Aβ oligomer species. Through this way, the non-pathological default neuronal processing of Aβ should be kept untouched and clean and not allow for pathological downstream processes like formation of aberrant Tau isoforms in neurons. The ultimate goal will be to protect adults at the age of 40 or even earlier by active vaccination with a suitable designed Aβ antigen, e.g., as described in Barghorn et al. (2016), which elicits permanently low levels of endogenous anti-Aβ oligomer directed antibodies.

To summarize, the BAD hypothesis explains negative clinical results of major Aβ-directed clinical studies so far and lays the ground for a paradigm shift for effective and selective Aβ oligomer-related clinical prevention and hopefully treatment concepts in AD.

## Author Contributions

HH wrote the manuscript.

## Conflict of Interest

The author declares that the research was conducted in the absence of any commercial or financial relationships that could be construed as a potential conflict of interest.
